# Clinical utility of liver function tests for resolution of metabolic dysfunction‐associated steatotic liver disease after weight loss in the Diabetes Remission Clinical Trial

**DOI:** 10.1111/dme.15462

**Published:** 2024-12-08

**Authors:** S. V. Zhyzhneuskaya, A. H. Al‐Mrabeh, C. Peters, A. C. Barnes, K. G. Hollingsworth, P. Welsh, N. Sattar, M. E. J. Lean, R. Taylor

**Affiliations:** ^1^ Magnetic Resonance Centre, Translational and Clinical Research Institute, Newcastle University Newcastle upon Tyne UK; ^2^ University Hospital of North Durham, County Durham and Darlington NHS Foundation Trust Durham UK; ^3^ Centre for Cardiovascular Science, Queen's Medical Research Institute, University of Edinburgh Edinburgh UK; ^4^ Human Nutrition Research Centre, Population Health Sciences Institute, Newcastle University Newcastle upon Tyne UK; ^5^ School of Cardiovascular and Metabolic Health, College of Medical Veterinary and Life Sciences, University of Glasgow Glasgow UK; ^6^ Human Nutrition, School of Medicine, Dentistry and Nursing, College of Medical Veterinary and Life Sciences, University of Glasgow Glasgow UK

**Keywords:** diet, liver disease, magnetic resonance imaging, non‐alcoholic steatohepatitis, type 2 diabetes

## Abstract

**Aims:**

Ectopic fat is reduced by effective weight management, but difficult to assess clinically.

**Methods:**

We evaluated paired data on 42 participants in the intervention group of the Diabetes Remission Clinical Trial (DiRECT) at baseline, 12 and 24 months after weight loss as indicators of liver fat content measured by 3‐point Dixon MRI.

**Results:**

Baseline liver fat was elevated at 13.0 [7.8–23.3]% with fasting plasma glucose 7.9 [7.1–10.1] mmol/L. Prevalence of baseline MASLD was 86.4%. After weight loss of 11.9 ± 1.2 kg (0–37 kg) at 12 months, remission of MASLD occurred in 74% and liver fat normalised for many (1.8 [1.2–5.2]%; *p* < 0.0001) as did fasting glucose (5.9 [5.5–7.2] mmol/L; *p* < 0.0001). Alanine aminotransferase (ALT) and gamma glutamyl transferase (GGT) decreased at 12 months by 38 [19–60]% (*p* < 0·0001) and 38 [16–53]% (*p* < 0.0001) respectively. The positive predictive value for decrease in liver fat, with baseline values of >40 IU/L, was 100% for ALT and 87.5% for GGT. As expected, change in liver fat correlated with change in ALT (*r* = 0.64; *p* < 0.0001), GGT (*r* = 0.38; *p* = 0.013), AST (*r* = 0.36; *p* = 0.018), fatty liver index (*r* = 0.50; *p* < 0.0001) and hepatic steatosis index (*r* = 0.44; *p* < 0.0001).

**Conclusion:**

Metabolic dysfunction‐associated steatotic liver disease, an important marker of ill‐health is improved by intentional weight loss. If enzyme levels are raised at baseline, following weight loss, changes in ALT and GGT usefully reflect change in liver fat content, with high positive predictive value. Monitoring liver enzymes can provide a simple way to assess change in liver fat following weight loss in day‐to‐day clinical practice.

**What‘s new?**

- Substantial weight loss had a potent effect on liver fat content leading to remission in 74% of those with MASLD at baseline.
- The proportion achieving remission of MASLD induced by dietary weight loss of 10–15 kg is similar to that seen with very large weight losses post‐bariatric surgery.
- Decreases in ALT and GGT following weight loss can be used to reflect change in liver fat as they have a high positive predictive value for normalisation of liver fat content when baseline levels are raised.
- Sequential measurements of liver enzymes in clinical practice can provide a simple means of assessing change in liver fat.

## 1 INTRODUCTION

Once considered to be an incidental finding of little clinical importance, metabolic dysfunction‐associated steatotic liver disease (MASLD), formerly known as non‐alcoholic fatty liver disease (NAFLD), is now an established indicator of morbidity and mortality. The overall prevalence of MASLD around the world is rising and estimated to be above 20%.^1^ An increase in liver fat is important in the aetiology of type 2 diabetes^2^, ^3^, ^4^ among whom the prevalence of MASLD is at least 70%.^5^, ^6^

There are currently no simple, reliable methods of measuring liver fat in primary care. Referral for liver ultrasound can provide an estimate but accurate quantification requires magnetic resonance imaging (MRI). Remission of type 2 diabetes is now a key therapeutic goal, and this requires normalisation of liver fat, by weight loss.^2^, ^7^, ^8^ Therefore, knowledge of return to normal liver fat levels would be valuable in clinical practice, both for motivation and monitoring of interventions. Knowledge of the possibility of remission of type 2 diabetes has alerted many participants to the presence and implications of MASLD. Liver enzyme measurement at a single time point is not reliable for assessing MASLD,^9^ though when seen in conjunction with elevated BMI, triglycerides and often HbA1c, they are often signalling excess liver fat.^10^ Furthermore, the major change in liver fat content which accompanies substantial weight loss might be accompanied by a clinically useful decrease in liver enzymes, serving as a pragmatic biomarker for liver fat reduction.^1^ Data are required to explore this possibility.

In the Diabetes Remission Clinical Trial (DiRECT) magnetic resonance measurement of liver fat content and LFT data were obtained in a trial subgroup^11^ permitting assessment of the relationship during weight loss. Additionally, the Fatty Liver Index^12^ and Hepatic Steatosis Index^13^ could be calculated. The present exploratory post‐hoc analysis was conducted to evaluate the predictive value of serial liver function tests and derived indices of MASLD during substantial weight loss, as judged against the gold standard of magnetic resonance measurement of liver fat.

## 2 RESEARCH DESIGN AND METHODS

### 2.1 Participants

Participants in the Tyneside cohort of DiRECT randomised to the weight loss management intervention were studied. Inclusion criteria were type 2 diabetes duration of less than 6 years, age 20–65 years, BMI between 27 and 45 kg/m^2^ and HbA1c ≥ 48 mmol/mol (≥6.5%) if on diet alone or HbA1c ≥ 43 mmol/mol (≥6.1%) if on treatment with oral hypoglycaemic agents. The recruited group (*n* = 59) was typical of early type 2 diabetes: age 52.5 ± 1.1 years; 50·9% men; weight 100.4 ± 2.3 kg; BMI 34.9 ± 0.6 kg/m^2^. Duration of diabetes since the diagnosis was 3.0 ± 0.2 years. Full data on liver fat and LFT were available on 59 participants at baseline and on 42 participants for paired data at baseline, 12 and 24 months.

Non‐Diabetic Controls (NDC, *n* = 25) were recruited to permit comparison with the DiRECT group matched for post weight loss weight and BMI, and age and sex (86.6 ± 3.0 kg; 29.7 ± 0.8 kg/m^2^, 55.8 ± 1.2 years; 52% men; Table 1). Oral Glucose Tolerance Tests were performed on this group to confirm normal glucose tolerance, and none had known personal or family history of type 2 diabetes.

**TABLE 1:** Summary of the change in weight and fasting metabolic parameters for type 2 diabetes group at baseline, 12 months and 24 months and for non‐diabetic control (NDC) group.

	Type 2 diabetes group (*n* = 42)			NDC (*n* = 25)
	Baseline	12 months	24 months	
Weight (kg)	98.3 ± 2.5††	86.4 ± 2.2***	90.7 ± 2.4***	86.6 ± 3.0
BMI (kg/m^2^)	34.2 ± 0.7†††	30.2 ± 0.7***	31.6 ± 0.7***	29.7 ± 0.8
HbA1c (mmol/mol) (%)	58.0 [53.3–63.8]††† 7.5 [7.0–8.0]†††	42.9 [38.3–53.6]***††† 6.1 [5.7–7.1]***†††	50.0 [43.0–61.0]*††† 6.7 [6.1–7.7]*†††	36.0 [32.0–38.0] 5.4 [5.1–5.6]
FPG (mmol/L)	7.9 [7.1–10.1]†††	5.9 [5.5–7.2]***†††	6.5 [5.7–7.9]**†††	5.1 [4.8–5.4]
FPI (pmol/L)	75.9 [51.1–113.0]†††	30.1 [20.2–54.2]***†	44.1 [18.2–59.2]***††	16.4 [10.0–37.2]
Liver fat (%)	13.0 [7.8–23.3]†††	1.8 [1.2–5.2]***	6.5 [3.0–9.0]***†††	1.9 [1.0–4.9]
ALT (units/L)	30.8 [22.1–38.1]††	15.8 [13.7–20.3]***	21.6 [17.0–26.0]**	18.5 [15.8–26.5]
GGT (units/L)	35.0 [24.5–51.5]†	22.0 [16.3–29.5]***	24.0 [18.0–33.5]***	19.5 [15.8–41.8]
AST (units/L)	19.3 [15.4–25.1]	16.4 [13.7–18.7]***	17.9 [15.2–19.3]**	Not available
Fasting cholesterol (mmol/L)	4.08 [3.53–4.90]†††	4.17 [3.42–5.07]†††	4.56 [3.93–5.56]**	5.30 [4.60–5.80]
Total fasting TG (mmol/L)	1.72 [1.17–2.22]††	1.19 [0.81–1.60]***	1.26 [0.91–1.75]***	1.10 [0.80–1.50]

*Note*: Data shown as mean ± SEM or median [IQ range]. Comparisons are shown for baseline to 12 months, and baseline to 24 months (***p < 0.001, **p < 0.01, *p < 0.05) and for type 2 diabetes group versus NDC at each time point (†††*p* < 0.001, ††*p* < 0.01, †*p* < 0.05).

### 2.2 Study protocol

The present study involves a subset of participants in the DiRECT intervention group, who followed an integrated weight management programme, with 12–20 weeks on 825–853 kcal/day liquid formula diet to induce weight loss, a 4–6 period of food reintroduction and then food‐based weight maintenance diet. Quantification of liver fat and LFTs was carried out at baseline, then at 12 and 24 months of follow‐up in DiRECT. The NDC group were studied on a single occasion.^7^

After fasting from 10:00 PM of the previous day all participants had liver fat measurement by MR and blood sampling. The morning medications were omitted on the day of the study and antidiabetic and antihypertensive treatments were then discontinued, being reintroduced if indicated from blood glucose and blood pressure monitoring. Participants were reviewed at their GP practice fortnightly until they had completed food reintroduction, then monthly to limit and address weight regain during the 2‐year follow‐up phase.

Ethical approval was obtained from the West of Scotland Research Ethics Committee (reference number: 13/WS/0314). DiRECT was registered on 20 December 2013, Current Controlled Trials number ISRCTN03267836.

### 2.3 Measurements and analytical procedures

Magnetic resonance images were collected by using a 3 Tesla Phillips Intera Achieva Scanner (Phillips, Amstelplein 2, 1096 BC Amsterdam, Netherlands). A standard 6 channel cardiac coil (Phillips) was used for taking images in most cases. In the circumstances of the large body habitus the so‐called Flexi Coil or four large surface coils (Phillips) were used instead. Liver fat data were gathered by using the Newcastle modification of the 3‐point Dixon MR scanning method as previously described using MATLAB software (Mathworks, Cambridge, UK).^14^ The Image J 1.43 software was used to define the Region‐of‐Interest (ROI) by utilising the Image J polygon tool.^15^ 5 ROIs for the liver were acquired and averaged, using the same anatomical area across every scan for each person sequentially. The inter‐scan Bland–Altman repeatability coefficients were 0.5% for the liver.^2^

Venous blood was sampled after MR scanning following the overnight fast from 10 pm of the previous day. Fasting plasma glucose was measured by using the glucose oxidase method (YSI glucose analyser, Yellow Springs Instrument Company, Yellow Springs, OH). Liver enzymes (ALT, GGT, AST) and HbA1C were analysed at the Institute of Cardiovascular and Medical Sciences, University of Glasgow (c311, Roche Diagnostics, Burgess Hill, UK). Fasting plasma insulin (FPI) was measured by ELISA (Mercodia, Uppsala, Sweden) at the Clinical Pathology Laboratory of Newcastle upon Tyne Hospitals NHS Trust.

Hepatic Steatosis Index (BMI, AST, ALT, diagnosis of type 2 diabetes)^12^ was calculated using MD App (https://www.mdapp.co/hepatic‐steatosis‐index‐hsi‐calculator‐357/) calculator. As a secondary objective, the BARD score for an assessment of the risk of hepatic fibrosis, based on BMI, AST/ALT ratio and the presence/ absence of type 2 diabetes,^16^ was calculated using MD Calc online calculator (https://www.mdcalc.com/bard‐score‐nafld‐fibrosis). Fib‐4 score could not be used as platelet count was not available.

Data were analysed using IBM SPSS statistical software (www.ibm.com). Normality of the data was examined using histograms and Shapiro–Wilk test. Paired data for 42 people with type 2 diabetes was analysed at all timepoints in comparison with baseline values and with NDC. Correction for multiple testing was not done. Normally distributed data were analysed using paired samples *t*‐test, independent samples *t*‐test or Pearson correlation test, and skewed data were analysed using Wilcoxon Rank test, Mann–Whitney *U*‐test, or Spearman Rank correlation test as appropriate. Data were presented as mean ± SEM or median with interquartile range [25th and 75th centiles].

Positive predictive values (PPV) of ALT and GGT for normalisation of liver fat (<5.6%) post weight loss were calculated in the diabetes group only (*n* = 42) at 12 months.

### 2.4 Results

**FIGURE 1:**
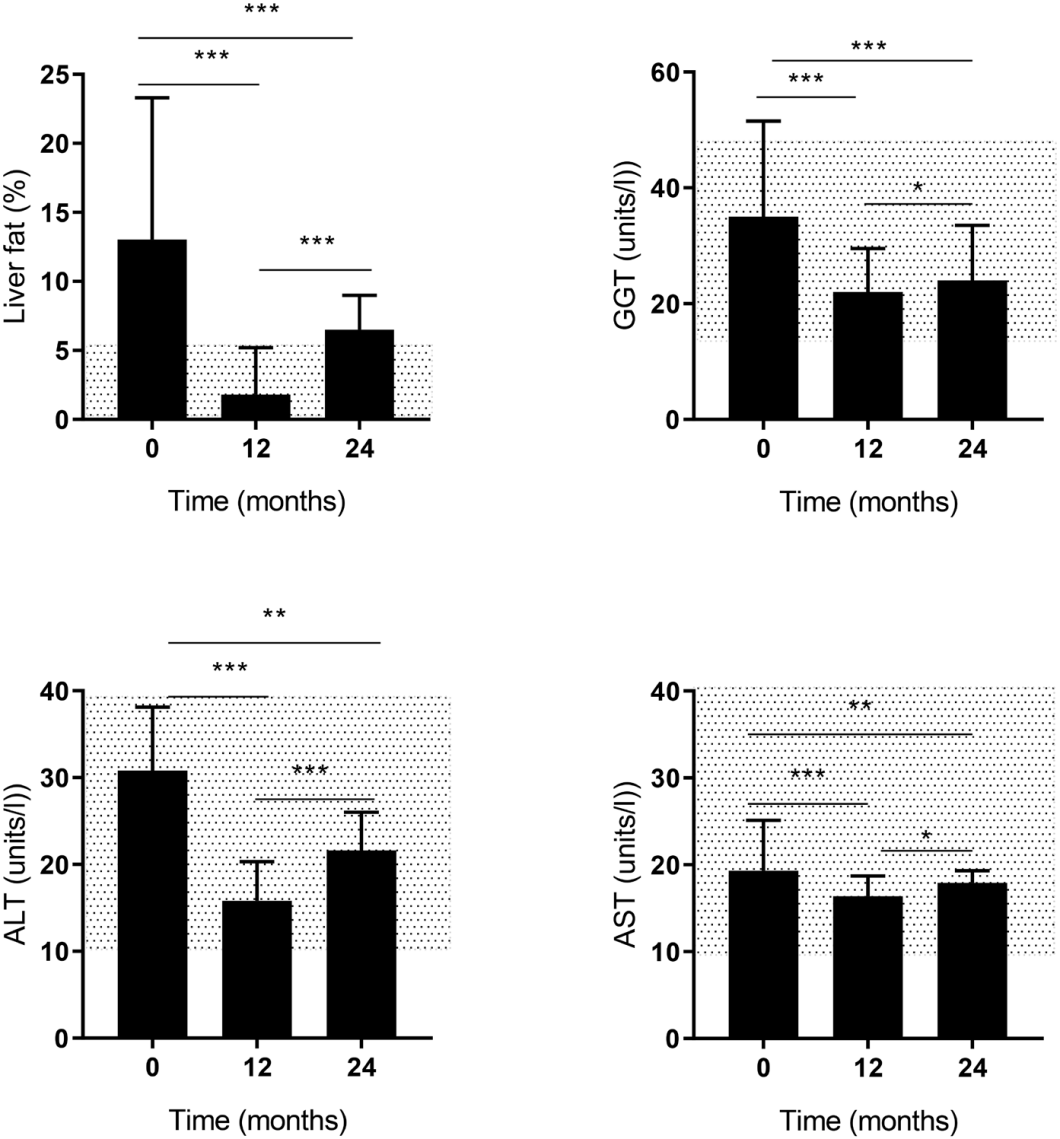
Changes in liver fat and liver enzymes in type 2 diabetes group at baseline, 12‐ and 24‐month follow‐up. This Figure shows liver fat, ALT, GGT and AST (median with interquartile range) in type 2 diabetes group at baseline, 12 and 24 months follow‐up (*n* = 42 at each time point). Statistical changes marked with horizontal lines between the time points (***p < 0.001, **p < 0.01, *p < 0.05). Shaded area represents normal ranges of the liver fat, ALT, GGT and AST respectively.

Mean weight loss at 12 months was 11.9 ± 1.2 kg (range 0–37 kg). Weight decreased from 98.3 ± 2.5 kg at baseline to 86.4 ± 2.2 kg (*p* < 0.0001) at 12 months and increased by 4.2 ± 0.6 kg to 90.7 ± 2.4 kg (*p* < 0.0001; Table 1) at 24 months. 33% (*n* = 14) of participants had maintained ≥10 kg weight loss at 24 months. Liver fat content was grossly elevated at baseline at 13.0 [7.8–23.3]% (upper level of normal 5.6%).^17^ It decreased to 1.8 [1.2–5.2]% at 12 months (*p* < 0.0001) becoming comparable with NDC (1.9 [1.0–4.9]%, *p* = 0.99). Change in liver fat correlated strongly with change in weight and indices of glucose control at 12 months (Figure 3). At 24 months, liver fat had increased to 6.5 [5.7–7.9]% (*p* < 0.0001) (Figure 1).

The baseline prevalence of MASLD (defined as above the upper limit of normal) was 86.4% (*n* = 51/59). It was similar in those with paired data at both time points (83.3%; *n* = 35/42) and the prevalence of MASLD declined to 21.4% (*n* = 9/42) at 12 months, becoming similar to NDC (24.0%; *n* = 6/25). With weight regain the prevalence of MASLD in the type 2 diabetes group rose between 12 and 24 months to 54.8% (*n* = 23/42).

Remission of MASLD (liver fat content below the 5.6% threshold) was achieved at 12 months in 26 of the 35 people with MASLD at baseline (74.3%) or 26 of the total 42 type 2 diabetes people (61.9%). At 24 months, remission of MASLD persisted in 14/35 (40%) or 14/42 (33.3%) of the whole group.

### 2.5 Liver enzymes

Baseline liver fat in the whole type 2 diabetes group (*n* = 59) correlated positively with ALT (*r* = 0.45; *p* = 0.0003) but less so with GGT (*r* = 0.23; *p* = 0.079).

*Alanine aminotransferase* (*ALT*) fell after weight loss by 38 [19–60]%: from 30.8 [22.1–38.1] IU/L at baseline to 15.8 [13.7–20.3] IU/L (*p* < 0.0001) at 12 months (Table 1). At 24 months, ALT had increased from 12‐month values to 21.6 [17.0–26.0] IU/L but remained significantly lower than baseline (*p* < 0.001). At both 12 and 24 months, ALT was not significantly different from NDC (18.5 [15.8–26.5] IU/L) (*p* = 0.088 and *p* = 0.308 respectively). When considered in relation to degree of weight loss, ALT decreased by 48 [32–61]% and 23 [14–54]%, in those losing >10 kg and <10 kg respectively at 12 months.

*Gamma glutamyl transferase* (*GGT*) decreased 38 [16–53]% from 35.0 [24.5–51.5] IU/L to 22.0 [16.3–29.5] IU/L ((*p* < 0.0001) at 12 months; Table 1) and remained steady to 24 months (24.0 [18.0–33.5] IU/L; *p* < 0·0001 vs. baseline). It became close to that of the NDC group (19.5 [15.8–41.8] IU/L) at 12 months (*p* = 0.674) and 24 months (*p* = 0.566). GGT fell by 52.0 [33.1–63.5] % (*p* < 0.0001) and 28.7 [12.3–37.2] % (*p* = 0.003) respectively with weight losses of >10 kg and <10 kg at 12 months.

*Aspartate aminotransferase* (*AST*) decreased between baseline and 12 months by 20.1 [3.8–39.0]% from 19.3 [15.4–25.1] IU/L to 16.4 [13.7–18.7] IU/L (*p* < 0.0001). It then remained steady at 24 months (17.9 [15.2–19.3] IU/L; *p* = 0.003 vs. baseline). AST fell by 22.8 [12.7–35.9]% at 12 months in the >10 kg weight loss subgroup (*p* < 0.0001) and by 11.9 [−10.5–39.1]% in those who lost <10 kg (*p* = 0.050).

**FIGURE 2:**
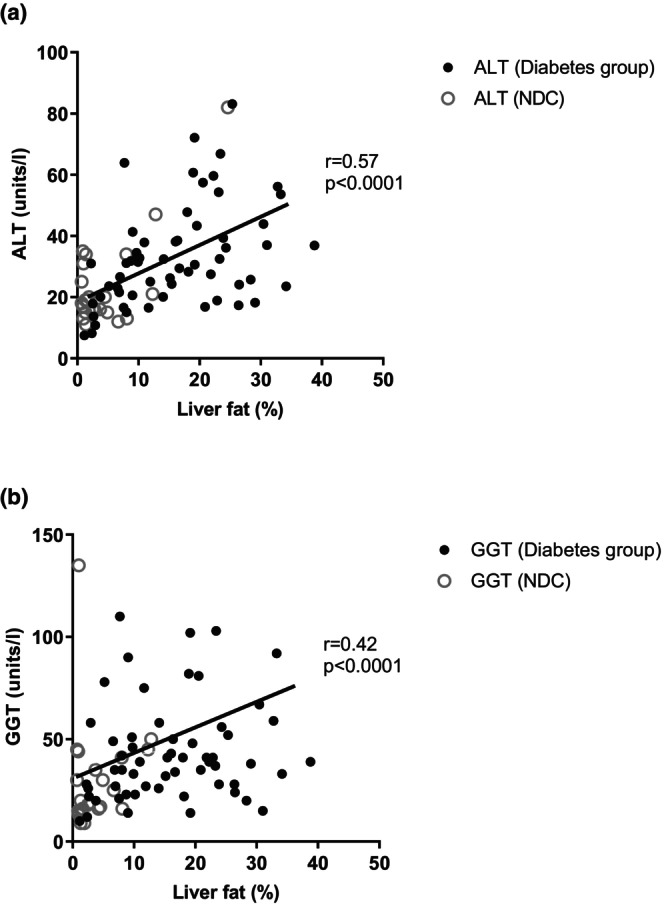
Relationship between liver fat and liver enzymes in the whole type 2 diabetes group at baseline and NDC combined. (Graph a) shows correlation between ALT (units/l) and liver fat (%) at baseline. (Graph b) shows correlation between GGT (units/L) and liver fat (%) at baseline.

**FIGURE 3:**
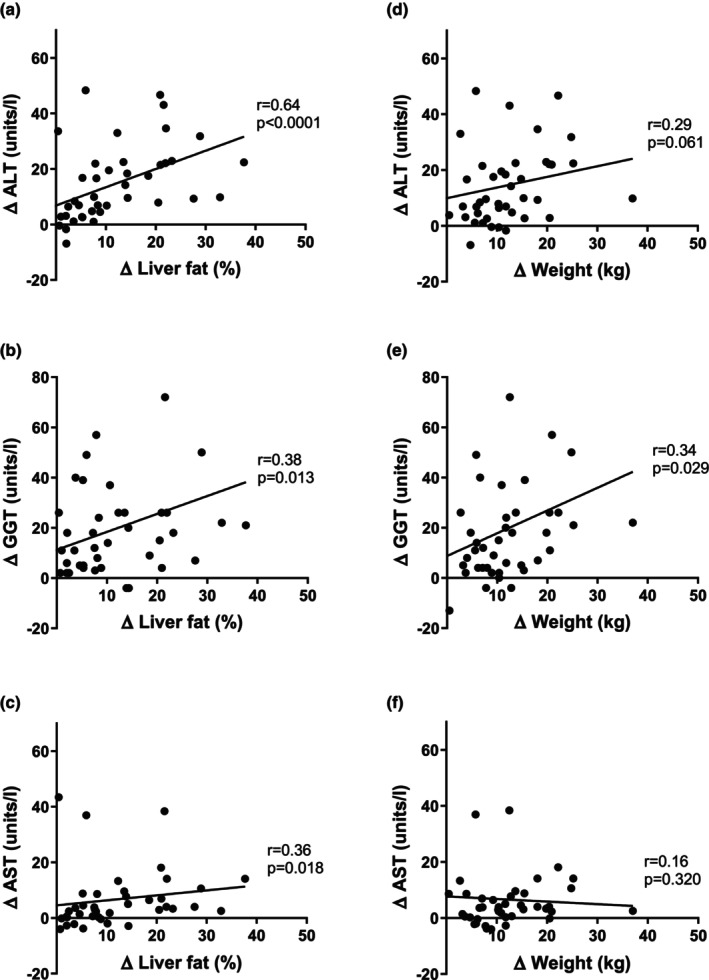
Relationship between changes in liver fat, liver enzymes and weight in type 2 diabetes group. (a, b, c graphs) show correlations between changes in liver fat and ALT, GGT, AST respectively in type 2 diabetes group (*n* = 42) post weight loss at 12 months. (d, e, f graphs) show correlations between changes in weight and liver enzymes in type 2 diabetes group (*n* = 42) at 12 months.

In the whole group of participants with and without type 2 diabetes at baseline, liver fat correlated with ALT (*r* = 0.57; *p* < 0.0001) and with GGT (*r* = 0.42; *p* < 0.0001) (Figure 2). Between baseline and 12 months, there were positive correlations between changes in liver fat and ALT (*r* = 0.64; *p* < 0.0001), GGT (*r* = 0.38; *p* = 0.013) and AST (*r* = 0.36; *p* = 0.018) (Figure 3a–c).

ANOVA was performed to examine the interactions between variables potentially affecting change in liver fat post intervention (changes in weight, BMI, ALT, AST, GGT, lipid oxidation, total plasma cholesterol, total plasma triglycerides). With liver fat as the dependent variable, there were associations only with fall in weight (*p* = 0.001), BMI (*p* < 0.001) and ALT (*p* = 0.001).

### 2.6 Indices of hepatic steatosis and hepatic fibrosis

**FIGURE 4:**
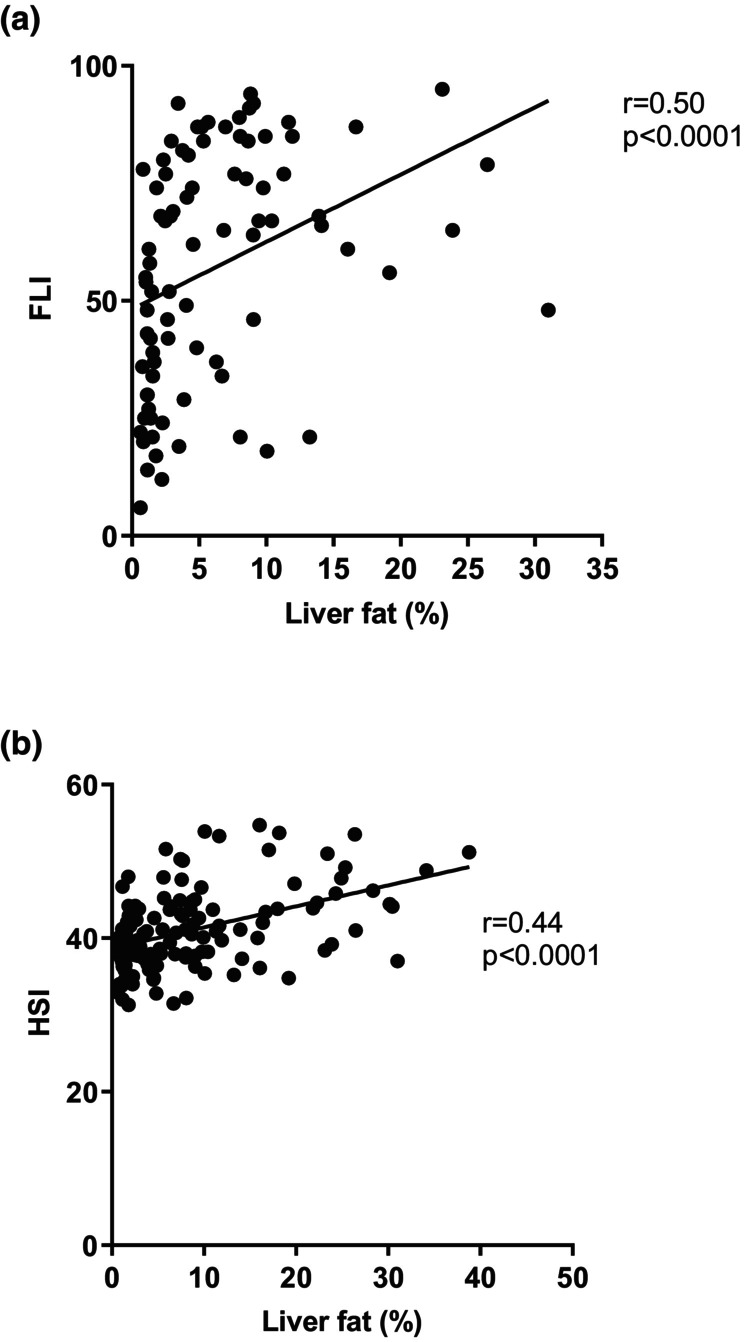
Relationship between liver fat and indexes of hepatic steatosis in type 2 diabetes group at all timepoints. (Graph a) shows correlation between fatty liver index (FLI) and liver fat (%). (Graph b) shows correlation between hepatic steatosis index (HSI) and liver fat (%).

The hepatic steatosis index decreased from 43.2 ± 0.8 at baseline to 39.7 ± 0.8 at 12 months (*p* < 0.0001) and remained steady at 24 months (40.2 ± 0.8). Change in weight correlated with change in hepatic steatosis index at 12 months (*r* = 0.65; *p* < 0.0001) as well as with absolute weight (*r* = 0.58; *p* < 0.0001), BMI (*r* = 0.91; *p* < 0.0001) and liver fat (*r* = 0.47; *p* = 0.002) (Figure 4), fatty liver index (*r* = 0.56, *p* = 0.001).

The BARD score of fibrosis was not changed at 12 months (2.7 ± 0.2 vs. 2.8 ± 0.2 at baseline, *p* = 0.800) but had improved at 24 months (2.4 ± 0.2; *p* = 0.041) compared both with baseline and 12 months values (*p* = 0.032).

### 2.7 Predictors of change in liver fat content

In the whole type 2 diabetes group, decreases in ALT and GGT after dietary intervention at 12 months had positive predictive value (PPV) for normalisation of liver fat content of 75.0% and 90.0% respectively.

Threshold effects of the scores were investigated. For those with a baseline ALT of >40 IU/L, PPV for normalisation of liver fat content after dietary intervention at 12 months was 100% with NPV of 33.3%. With levels >25 IU/L at baseline, PPV was 91.7% with NPV of 16.7% (Table 2). For GGT the optimal threshold for detecting the normalisation of liver fat content after dietary intervention at 12 months was >35 IU/L at baseline resulting in a PPV of 92.3%.

**TABLE 2:** Positive predictive values (PPV) between decrease in ALT and GGT and normalisation of liver fat post weight loss in diabetes group (*n* = 42) at 12 months with respect to different baseline cut‐offs for the named liver enzymes.

	PPV (%)
*ALT* (*units*/*L*)	
>40	100.0
>35	85.7
>30	90.0
>25	91.7
*GGT* (*units*/*L*)	
>40	87.5
>35	92.3
>30	84.6
>25	85.7

PPV for the change in hepatic steatosis index (HSI) detecting the normalisation of liver fat at 12 months for the whole type 2 diabetes group was 93.3%.

## 3 DISCUSSION

These data on people with type 2 diabetes and BMI >27 kg/m^2^ (mean BMI 34.9 kg/m^2^) at baseline demonstrate that sequential measurement of individual liver enzymes allowed prediction of normalisation of liver fat levels with mean weight loss 11.9 kg at 12 months or 74% of those with MASLD at baseline. If baseline ALT was raised >40 IU/L, normalisation at 12 months after weight loss had a positive predictive value of 100%. The positive predictive value for normalisation of liver fat post weight loss was 75% and 90% for ALT and GGT respectively, although only change in ALT was independently associated with change in liver fat on multivariate analysis in conjunction with a fall in weight and BMI. ALT also had the strongest correlation with weight change of all markers tested. Use of these widely available tests permit diagnosis of remission of MASLD in routine clinical practice. Calculation of the hepatic steatosis index did not provide additional benefit.

Liver function tests have a wide variability in apparently healthy people under many metabolic and environmental influences but remain fairly constant within any individual. They rise when there is any injury to liver tissue, and intrahepatic fat is such a causative factor in part via local, low‐grade inflammation. Fatty liver is a key element in the aetiology of type 2 diabetes^3^, ^4^ but difficult to assess in primary care. Neither ALT nor GGT have high reliability individually as point estimates to confirm or exclude the presence of MASLD^9^, ^18^ but in type 2 diabetes cohorts, 57% have at least one and 27% have two or more abnormal LFT tests.^19^ Elevated GGT is well recognised to be associated with type 2 diabetes, as well as being sensitive to excess alcohol intake, and a predictor of fibrosis/cirrhosis.^20^ Compared with type 1 diabetes, people with type 2 diabetes exhibit a higher prevalence of elevated ALT (22.9 vs. 5.3%, *p* < 0.01) and GGT (23.7 vs. 10.5%, *p* < 0.01).^21^ This is consistent with observations from a four‐country study of detected MASLD in which the one which uses more imaging for MASLD diagnosis reports the highest prevalence of MASLD.^22^ However, the clinical importance of the present study lies in demonstration of the clinical utility of sequential decrease in simple liver enzymes to assess change in hepatic steatosis, reflecting the reverse of the sequential increase seen during overfeeding studies.^23^, ^24^ These changes are reflected in the 5‐year follow‐up data from DiRECT.^25^ Full clinical assessment involves measurement of plasma triglyceride and HbA1c levels which, as noted above, decline in step with falling liver fat levels, further supporting a reduction in ectopic fat as we have previously argued.^10^

Achieving a complete return to normal of liver fat content by many participants after weight loss underscores the relationship of MASLD with overnutrition in susceptible individuals, particularly those who exhibit prediabetes or type 2 diabetes.^19^, ^24^ The clinical utility of tracking the return to normal using liver function tests or derived indices is relevant not only to monitoring liver health during remission of type 2 diabetes but also to the wider management of people with presumed or proven MASLD. The Hepatic Steatosis Index (HSI)^13^ is also imprecise for diagnosis of MASLD as a point estimate, but like plasma liver enzymes can be used for sequential demonstration of change after weight loss, especially when baseline levels are raised. However, it requires a calculation, is time consuming in clinical practice, and fails to offer superior predictive power over sequential plasma ALT levels.

Importantly, in diagnosed MASLD, with or without type 2 diabetes, many individuals have ALT and GGT levels in elevated or the ‘high‐normal’ range.^9^ The upper level of ALT which may be regarded as ‘normal’ is open to question as the thresholds are based upon the values found in general populations which include many individuals who are overweight, possibly with prediabetes, and who have undiagnosed fatty liver disease. These findings are in line with ALT levels seen in participants with NAFLD in a recent European wide study of the prevalence of the diagnosed MASLD.^26^ Derivation of a truly normal range for ALT and GGT based on a population shown not to have MASLD would allow reassessment of the value of point measurements of these enzymes for diagnosis of MASLD.

The proportion achieving remission of diabetes, and of MASLD, induced by dietary weight loss of 10–15 kg is like that seen with very large weight losses post‐bariatric surgery. Up to 85% of those losing 25% of their initial body weight had remission of MASLD 1‐year post‐surgery.^27^ A weight loss of 13.5% after either bariatric surgery or dietary induced weight loss achieves a maximum decrease in liver fat.^28^ This was confirmed in a meta‐analysis of bariatric surgery studies,^29^ with remission of diabetes of around 83% at 2 years after bariatric surgery with weight losses up to 35%. It was similar (83% at 2 years) after non‐surgical weight loss >10 kg in DiRECT.^7^ Importantly, reversal of hepatic fibrosis has been detected using transient elastography after bariatric surgery.^30^ In the present study, the observation of decrease of the BARD score at 2 years is notable, suggesting potential improvement in this serious condition after dietary weight loss. Further research to evaluate this finding is warranted.

Limitations of the present study must be considered. The intensive diet intervention and real‐time primary care‐based setting of DiRECT precluded liver biopsies to confirm a relationship with hepatocyte ballooning, liver inflammation or fibrosis.^31^ LFTs were not measured in all participants immediately after the 3–5‐month weight loss period, when maximum weight change occurred. However, at 12 months when weight was relatively stable after some early regain, there was a clear correlation between extent of weight loss and decrease in liver fat from baseline. The participants on DiRECT reflected the populations studied (typical white European ethnicity with mean BMI 34.9 kg/m^2^) but it cannot be assumed that plasma liver enzymes will have the same relationship in people of differing ethnicity. In DiRECT, known alcohol or other substance abuse was an exclusion although we cannot rule out undeclared prior high consumption. However, we observed the strongest relationships for ALT rather than GGT which would be expected to change more with fall in alcohol consumption.

In summary, following weight loss, decreases in ALT, GGT and hepatic steatosis index have a high positive predictive value for normalisation of liver fat content when baseline levels are raised. In day‐to‐day clinical practice, sequential monitoring of liver enzymes following intentional weight loss can provide a simple means of demonstrating reduction in liver fat.

## AUTHOR CONTRIBUTIONS

S. V. Zhyzhneuskaya performed the clinical work, analysed the data and co‐wrote the manuscript. A. H. Al‐Mrabeh performed the laboratory work and edited the manuscript. C. Peters performed clinical work and edited the manuscript. A. C. Barnes trained and mentored practice nurses and edited the manuscript. K. G. Hollingsworth developed methodologies and edited the manuscript. P. Welsh performed the laboratory work and edited the manuscript. N. Sattar contributed to discussion and reviewed the manuscript. M. E. J. Lean contributed to discussion and to reviews and editing of the manuscript. R. Taylor conceived the project, oversaw data analysis and co‐wrote the manuscript.

## FUNDING INFORMATION

This study was funded by Diabetes UK as a Strategic Research Initiative (award number 13/0004691).

## CONFLICT OF INTEREST STATEMENT

RT reports grants from Diabetes UK, and lecture fees from Novo Nordisk, Lilly and Nestle Healthcare. SZ reports the funding of the execution of the trial work by Diabetes UK. KGH reports funding of the execution of the work by Diabetes UK with no personal remuneration received. NS reports grants and personal fees from Boehringer Ingelheim; personal fees from AstraZeneca, Eli Lilly, Sanofi and NovoNordisk. AB reports personal speaker fees for local HCP education events from Napp pharmaceuticals, Novo Nordisk UK, Lily UK, TREND UK. MEJL reports personal fees from Counterweight, Nestle, Eli Lilly and Novo Nordisk, not related to the present work. PW reports grants from Roche Diagnostics, AstraZeneca, Novartis, Boehringer Ingelheim. AA‐M reports a grant from Diabetes UK to conduct the Re‐TUNE study. All other authors have no conflicts of interest.

## Data Availability

The data that support the findings of this study are available on request from the corresponding author. The data are not publicly available due to privacy or ethical restrictions.
